# The Chinese carpet flap for anterior cranial fossa reconstruction in cases of depressed frontal fractures: 10 years’ experience at a tertiary referral center

**DOI:** 10.1007/s10143-026-04273-2

**Published:** 2026-04-18

**Authors:** Eman H. Salem, Eslam A. Elsery, Abdallah Aljijikly, Mohamed Serageldin, Mohammad Mahdi Bagheri Asl, Ahmed Abdelwahab

**Affiliations:** 1 Department of Otolaryngology – Head and Neck Surgery, Mansoura University, Mansoura, Egypt; 2 Manchester program, Faculty of Medicine, Mansoura University, Mansoura, Egypt; 3 Department of Neurosurgery, Tabriz University of Medical Science, Tabriz, Iran; 4 Department of Neurosurgery, Mansoura University, Mansoura, Egypt

**Keywords:** Frontal sinus fractures, Cranial bone fractures, Nasofrontal outflow tract, Pericranial flap, Skull base reconstruction, Mucocele

## Abstract

Frontal sinus fractures account for 5–15% of facial injuries and endanger adjacent vital structures. We retrospectively analyzed 89 depressed frontal sinus fractures reconstructed with a pericranial flap (PCF) harvested in 29 of them at a tertiary center between 2014 and 2024 (MFM.IRB.ID code: R.25.08.3314). Most patients were young men (mean 18.3 ± 4.1 years); mechanisms included falls (42.7%) and motor-vehicle collisions (39.3%). Fracture types were isolated anterior table (*n* = 54), combined anterior/posterior tables (*n* = 35), and nasofrontal outflow tract (NFOT) involvement (*n* = 8). Using a bicoronal approach, we cranialized NFOT-disrupted sinuses, repaired dura in 33 cases, and rebuilt orbits as required. The vascularized “Chinese-carpet” PCF was split or folded to achieve multilayer anterior skull-base coverage without donor-site morbidity. Forehead projection improved by 3.4 ± 1.7 mm (range 2.9–16.8 mm). Over a 39.2-month mean follow-up, no cerebrospinal fluid leaks occurred. Minor complications included transient alopecia (*n* = 33), scalp paresthesia (*n* = 57), and five mucoceles managed conservatively. Our decade-long experience supports the PCF as a versatile, dependable option for complex frontal sinus reconstruction; larger multicenter studies should confirm its long-term efficacy.

## Introduction

Frontal sinus fractures are rare, accounting for 5–15% of all facial fractures in adult craniomaxillofacial trauma. However, they harbor significant morbidity and mortality because of their proximity ID="Q2" to vital structures [1]. They typically occur as a result of high-velocity blunt trauma, such as motor vehicle accidents, followed by assaults and sports-related injuries, and they are usually [2]. Although three-dimensional reconstructions can be beneficial, the sagittal, coronal, and associated with other facial fractures [3]. High-resolution computed tomography (HRCT) scanning is the modality of choice for evaluating frontal sinus injuries axial sections are individually valuable for evaluating different anatomical regions of the frontal sinus. Utilizing sagittal and coronal cuts can be effective in evaluating nasofrontal outflow tract (NFOT) involvement [4, 5].

Fractures of the posterior table are typically corrected to prevent intracranial complications, while fractures of the anterior table are treated to correct cosmetic deformity. Additionally, operative intervention for NFOT injuries aims to prevent delayed complications of frontal mucocele, or mucopyocele [6, 7]. Different approaches (either open or endoscopic) and reconstruction materials are available (such as titanium mesh and other alloplastic materials), but there is no agreement on which one should be considered as the preferred treatment modality [8, 9]. The pericranial flap (PCF) is an example of soft tissue flaps described in the literature that hold good postoperative outcomes in reconstruction [10, 11].

Herein, we present our institutional experience and elaborate a detailed algorithm for managing frontal sinus fractures to facilitate decision making and maximize the long-term outcomes. With particular focus and a meticulous description of different scenarios on how to tailor the PCF as a diverse and versatile option for ACF reconstruction in three cases of depressed frontal fractures.

## Materials and methods

This retrospective study included all patients with depressed frontal fractures presenting to the Mansoura University maxillofacial unit between January 2014 and December 2024. Mansoura Faculty of Medicine institutional review board approval was obtained (MFM.IRB.ID: R.25.08.3314). Inclusion criteria encompassed patients with depressed frontal fractures (anterior +/- posterior table) with or without associated facial fractures. Patients who were managed conservatively, endoscopically, or missed follow-up were excluded. Data on the participants was collected, including age, gender, etiology of injury, type of fracture, associated facial bone fractures, repair approach, techniques, and material.

**Fig. 1:**
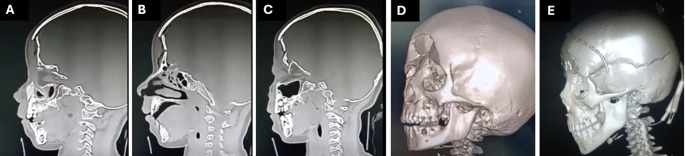
CT scan of depressed frontal sinus/bone fracture; **A, B, C**. sagittal cuts showing fractured orbital roof in A and C with entrapped bony spicules inside the orbit in C, fracture ethmoidal roof in B; **D, E** preoperative versus postoperative 3D CT scan showing improved projection after surgery

Preoperative computed tomography (CT) of the facial bones (coronal, axial, sagittal, and three-dimensional (3D) views) was done, meticulously reviewed to evaluate for cranial, ophthalmological, or NFOT involvement. It was compared with postoperative radiology (axial and sagittal) to assess improvement in the projection (Fig. 1). However, in cases of comminuted fractures where reduction of the small bony spicules was not possible and a soft tissue graft (fat or muscle) was used instead to restore the premorbid forehead contour, comparison of the pre and postoperative photographs was more realistic. External images were obtained with a high-resolution digital camera (Nikon, USA; Melville, NY, USA) taken by the same investigator.

Surgical treatment was carried out within 10 days after the trauma unless there was evidence of traumatic CSF leak, in which case, surgery was done within 2 weeks of failed conservative measures (carbonic anhydrase inhibitors + diuretics). The goal of surgery was to correct the cosmetic deformity, restore the premorbid forehead contour, correct any complication, and resolve the leak by using the PCF as a valid reconstructive material.

### Surgical steps

After initiation of general anesthesia, the nasal cavity was endoscopically examined to evaluate the NFOT and exclude any obstruction using 0-, 30-, 45-, and 70-degree, 18-cm length 4 mm diameter rod-lens endoscopes linked to a high-definition camera, and monitor (Karl Storz Endoscopy America; Redondo, California). Then, a forced duction test was performed to determine any entrapment of the extraocular muscle, if there is any orbital involvement.

### Pericranial flap (PCF) (Chinese carpet)

**Fig. 2:**
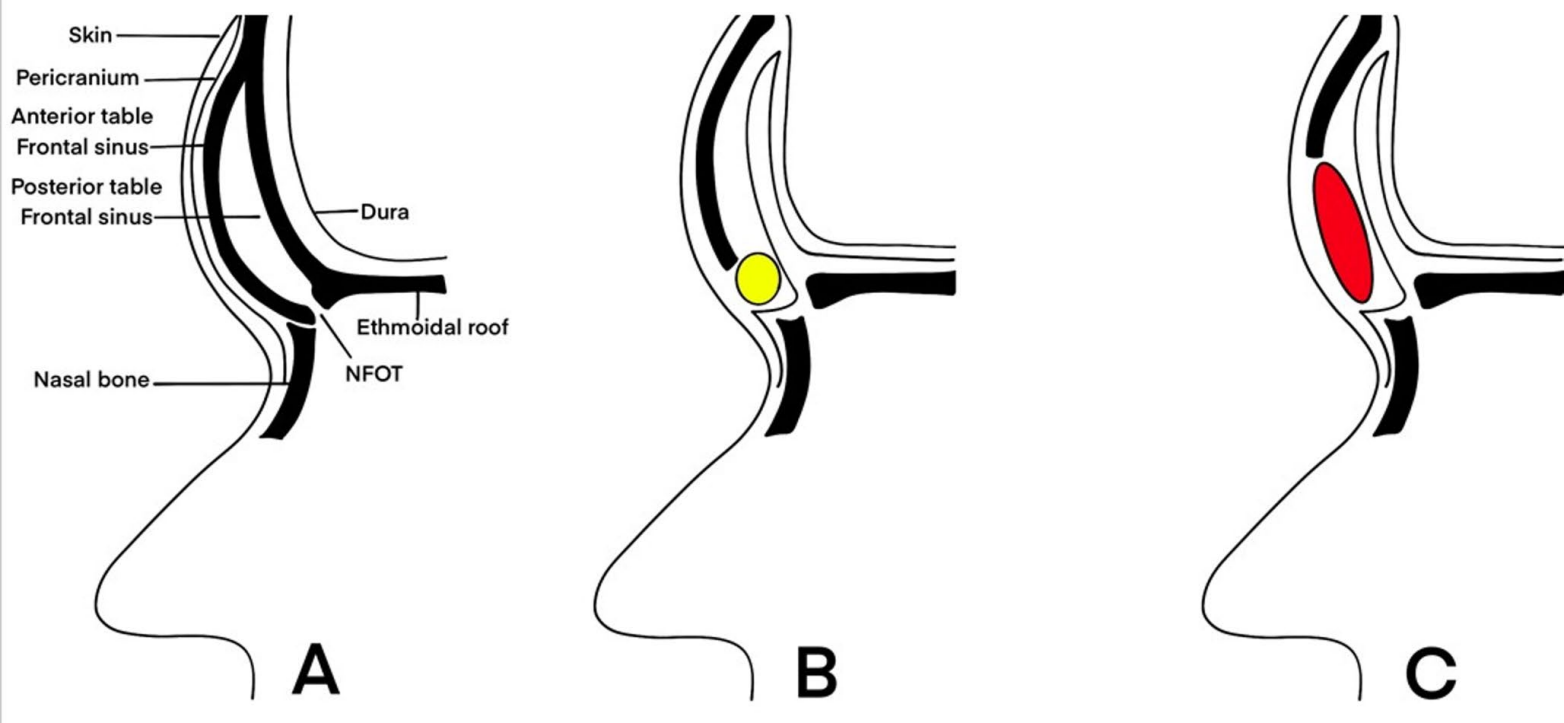
Visual illustration describing how to fold the PCF as a Chinese carpet for ACF reconstruction (**A.** before cranialization; **B. **cranialization+ PCF with fat graft; **C. **cranialization+ PCF with muscle graft)

With the use of a standard bicoronal incision, a conventional skin flap was dissected anteriorly in the subgaleal plane to the level of the supraorbital rims to adequately expose the frontal bone. Once the anteriorly based PCF was designed, its borders were incised sharply using the superior temporal lines as lateral limits of dissection. The posterior edge of the flap was incised in the region of the vertex to provide adequate flap length. Then the flap was gently elevated from the underlying calvarium and left pedicled mainly on the supratrochlear and supraorbital bundles. The PCF was draped over the denuded frontal sinus floor as well as over the exposed anterior cranial fossa (ACF) floor to cover any bony defects. Once the flap was tucked posteriorly under the inferior aspect of the retracted frontal lobes, it was then reflected superiorly so that it rested on the anterior frontal lobe dura matter. This folding of the flap on itself provided an additional layer of flap coverage between the frontal lobe dura and the damaged frontal bone (Fig. 2). In some cases where the central pedicle of the PCF was jeopardized by the fractured bones, splitting the flap and pedicling it laterally on superficial temporal artery was done (either unilaterally or bilaterally). The bony fragments were then fixed in place with non absorbable sutures or miniplates and screws. In cases where a fat/muscle graft were used to compensate for small/large bony defect , an overcorrection is usually preferred to guard against possible subsequent shrinkage of the graft. Finally, good closure of the periosteum and the skin was done in 2 layers with a 0.3 Vicryl and a subcuticular or interrupted Prolene, respectively. A negative suction drain was left in place for five days together with a tight bandage.

Postoperatively, patients were encouraged to frequently use ophthalmic antibiotic ointment and fomentations to minimize the edema and ecchymosis. Careful observation in the first 48 h was emphasized to early detect any frontal lobe manifestations resulting from the retraction intraoperatively. Oral antibiotics, antiedematous drugs (trypsin/chemotrypsin combinations), and analgesics were prescribed in the first postoperative week. The follow-up regimen was designed to be conducted twice per month in the first two postoperative months and monthly for the next six postoperative months, and photos were collected for the purpose of comparison.

### Statistical analysis

Descriptive statistics were calculated using SPSS (Statistical Package for Social Sciences) version 15. Qualitative data were presented as frequency and percentage. Quantitative normally distributed data were presented as mean ± standard deviation (SD). The Student T-test was used for the comparison of continuous variables.

### Ethical consideration and study registeration

Written informed consent for publication of surgical images and information was obtained from the parents or legal guardians of the pediatric patients included in our case presentations. When appropriate, assent was also obtained from the children, respecting their developing autonomy. The patients and their parents were informed about the purpose of publication, potential risks of identification, and the inability to withdraw consent after publication. All images were anonymized by removing direct identifiers and metadata, and by cropping or masking identifiable features to protect patient privacy.

This study was retrospective and did not involve a prospective clinical trial. The technique evaluated in this work is an established clinical practice; however, there is currently no consensus in the literature regarding its optimal use.

#### Clinical trial number

Not applicable.

## Results

One hundred thirty-seven cases of depressed frontal fractures were detected within the study period. Out of the 137 cases, 29 were managed conservatively, 12 were managed endoscopically, and 7 cases missed follow-up, resulting in a total of 89 cases included in the study.

Of the participants included, 54 patients had anterior table fractures, the remaining 35 had combined anterior and posterior table fractures, and only 8 patients had associated NFOT involvement necessitating cranialization. Associated facial fractures were encountered as zygomaticomaxillary complex (ZMC) fracture in 12 patients, fractured nasal bone in 10 patients, nasoethmoidorbital (NEO) fracture in 7 patients, and orbital blowout fracture in 5 patients. The most common causes of frontal bone fracture among all 89 patients were falls [38 patients (42.7%)] and motor vehicle accidents [35 patients (39.3%)], followed by assault (13.4%) and sports injury (4.5%). The mean age was 18.30 ± 4.12 years, and 76.4% were males.

Preoperative examination revealed 72 patients had external facial deformity, 29 had cerebrospinal fluid (CSF) leak (23 presented in the first week and 6 patients presented later), 18 patients had pneumocephalus, 26 had intracranial hemorrhage with disturbed GCS (14 had extradural, 9 had subdural, and 3 had intracerebral hemorrhage), and none presented with signs of meningitis. Preoperative enophthalmos was noticed in 11 cases; nine cases had diplopia, orbital ecchymosis (*n* = 23), and emphysema (*n* = 8), but there were no reported cases of ruptured globes, changes in vision, blindness, or orbital apex syndrome.

**Fig. 3:**
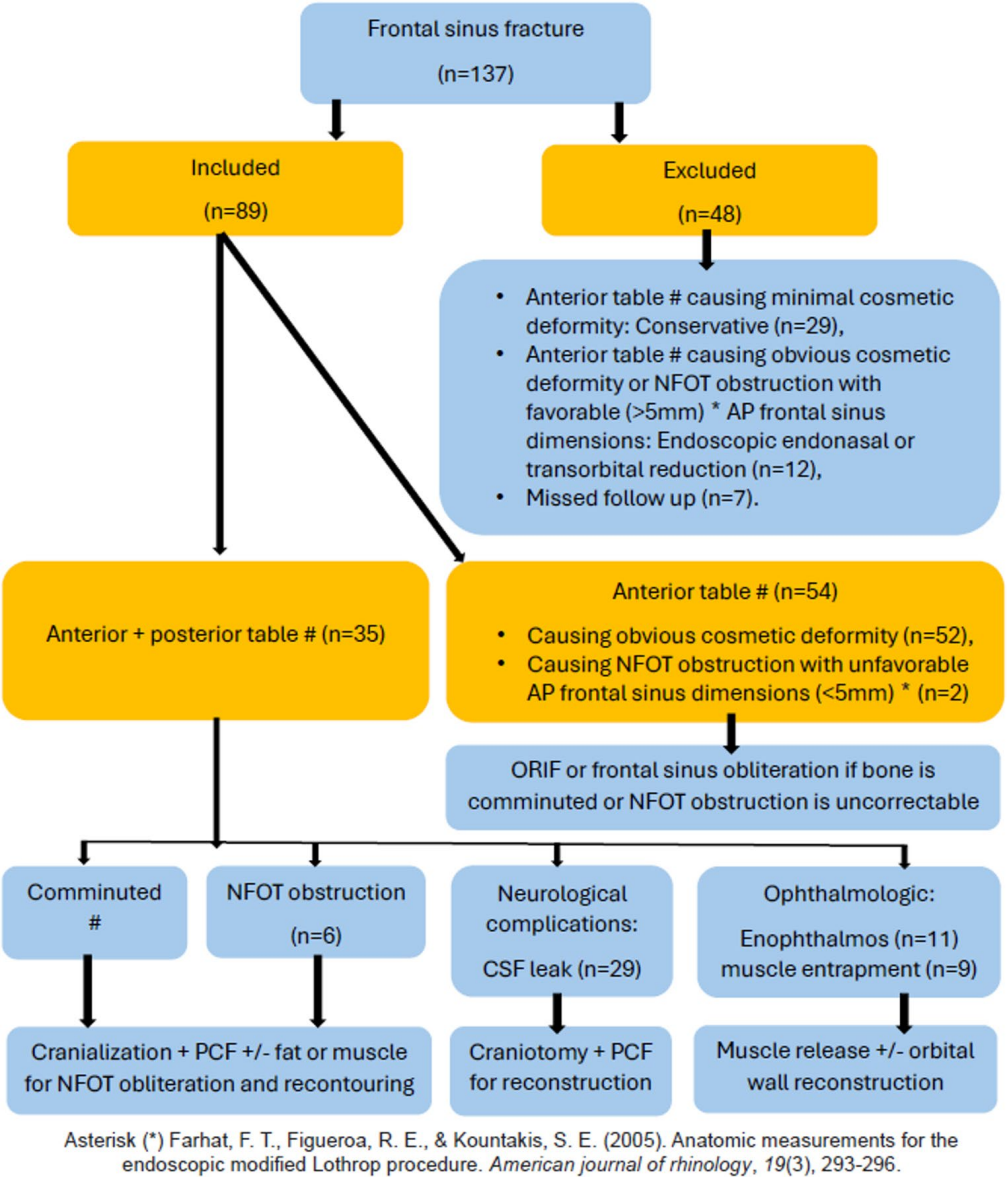
Algorithm illustrates how to select the appropriate approach for managing frontal sinus/bone fractures [12]

Management was tailored individually, where the included cases were subjected to open reduction and internal fixation through a bicoronal incision. They were either managed with a craniotomy (*n* = 61) or a split calvarial graft (*n* = 28). Dural repair was done for 33 patients, enforced with a pericranial flap in 29 of them. All patients who had evidence of NFOT involvement underwent cranialization with recontouring to the premorbid forehead projection using fat/muscle graft if there is a remaining bony defect or the comminution of the bone is impossible to be reduced. Figure 3 represents an algorithm illustrating how to select the appropriate approach in managing frontal sinus fractures.

The mean improvement in the forehead depression at one-month postoperative follow-up was 3.4 ± 1.7 mm (range 2.9–16.8 mm). No recurrence of CSF leak (*n* = 29) was reported in the follow-up period (mean 39.2 ± 4.7 months). All cases presented with diplopia reported correction, and the improvement was confirmed by ophthalmological consultation performing the Goldmann perimeter and Lancaster test. No major postoperative complications were detected apart from alopecia (*n* = 33), scalp paresthesia (*n* = 57), mucocele (*n* = 5), epiphora (*n* = 2), and meningitis (*n* = 2). Table 1 summarizes the demographic, clinical, and operative data of the patients.

**Table 1:** Summary of demographic, clinical, and operative data of studied population

Age	*N* = 89	%
	18.30 ± 4.12 years	(range 13–61 years)
Sex • Male • Female	68 21	76.4% 23.6%
Site of the fracture • Anterior table • Combined anterior and posterior tables	54 35	60.7% 39.3%
Associated facial fractures • ZMC • Nasal bone • NEO • Medial orbital blowout • Inferior orbital blowout	12 10 7 3 2	13.4% 11.2% 7.8% 3.4% 2.3%
Mode of trauma • Falls • Motor vehicle accidents • Assault (human and animals) • Sports injury	38 35 12 4	42.7% 39.3% 13.4% 4.5%
Neurological manifestations • External facial deformity • CSF leak • Pneumocephalus • Intracranial hemorrhage • Meningitis	72 29 18 26 0	80.9% 32.6% 20.2% 29.2% 0%
Ophthalmological manifestations • Enophthalmos • Diplopia • Orbital ecchymosis • Orbital emphysema • Rupture globe, Blindness	11 9 23 8 0	12.4% 10.1% 25.8% 8.9% 0%
Forehead projection improvement	3.4 ± 1.7 mm	(range 2.9–16.8 mm)
Mean follow-up	39.2 ± 4.7 months	(range 15–97 months)
Postoperative complications • CSF leak recurrence • Alopecia • Scalp paresthesia • Mucocele • Epiphora • Meningitis	0 33 57 5 2 2	0% 37.1% 64% 5.6% 2.3% 2.3%

### Case presentation

#### Case 1

**Fig. 4:**
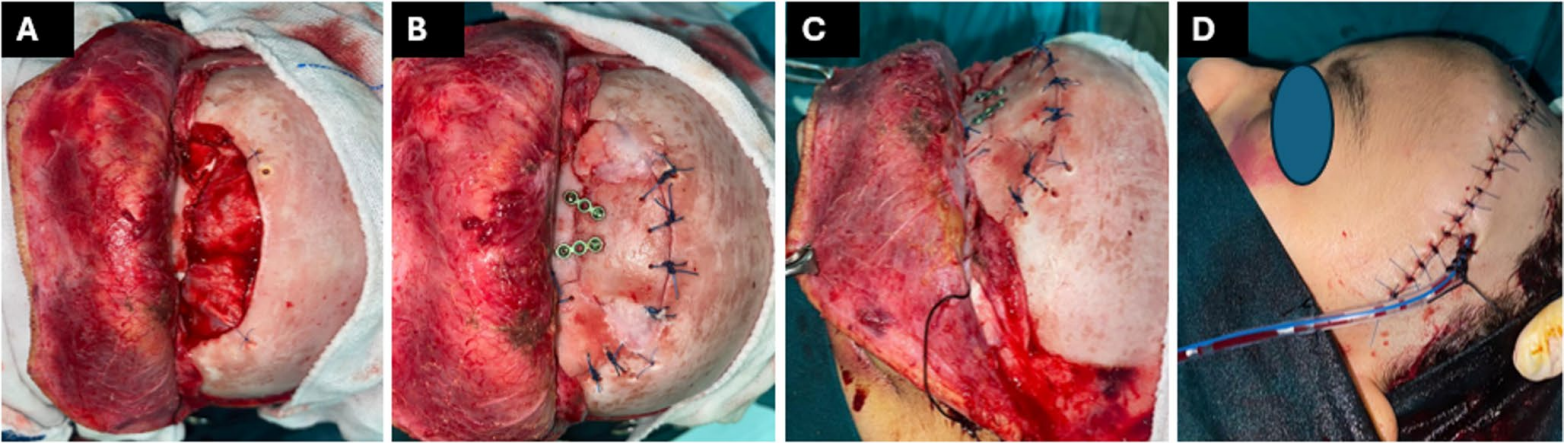
7-year-old girl, horse-kick injury, comminuted frontal sinus fracture repaired through a bicoronal approach. **A**. Craniotomy with removal of fractured segments and insertion of bilateral inferiorly based, laterally-pedicled PCF for ACF floor reconstruction; **B**. after plating with low-profile titanium miniplates and stitching the Craniotomy in place; **C**and** D**. Lateral views showing improved projection

A 7-year-old female patient, kicked by a horse, was presented to the emergency room (ER) with a forehead swelling, bilateral raccoon eyes, and limited adduction of the right eye. CT revealed a comminuted frontal fracture (anterior and posterior table) in an underpneumatized frontal sinus, a fractured cribriform plate, both orbital roofs, mild pneumocephalus, and a small epidural hematoma. The patient was prepared for surgery with a multidisciplinary collaboration between otolaryngology, ophthalmology, and neurosurgery. After performing a forced duction test to exclude orbital muscle entrapment, the procedure started by doing a bicoronal incision followed by raising an inferiorly based, superficial temporal artery laterally-pedicled PCF (since the supratrochlear/supraorbital bundles were jeopardized by the trauma). After exposure of the fractured bone, it was gently taken out as a frontal flap for evacuation of the hematoma. With mild frontal lobe retraction, the PCF was split into 2 sides, spread in a multilayer fashion to cover the ACF floor, and held in place with small pieces of gel foam, then the dura was stitched to the surrounding intact bone to obliterate any potential space in a trial to avoid recollection of the hematoma. The fractured pieces were fixed together with 1.5 mm Titanium miniplates and screws, and the frontal flap was secured in place with 1.0 Prolene stitches all around. The bicoronal flap was closed in 2 layers with an inner 3/0 vicryl and an outer 4/0 Prolene, the drain was removed on postoperative day 5, and the stitches were removed after 10 days. The patient was maintained on measures to decrease the ICP and protect against meningitis (3rd generation cephalosporin, diuretics, and acetazolamide) for 2 weeks. The follow-up period (18 months) went uneventfully with no CSF leak, meningitis, or other complications (Fig. 4).

#### Case 2

**Fig. 5:**
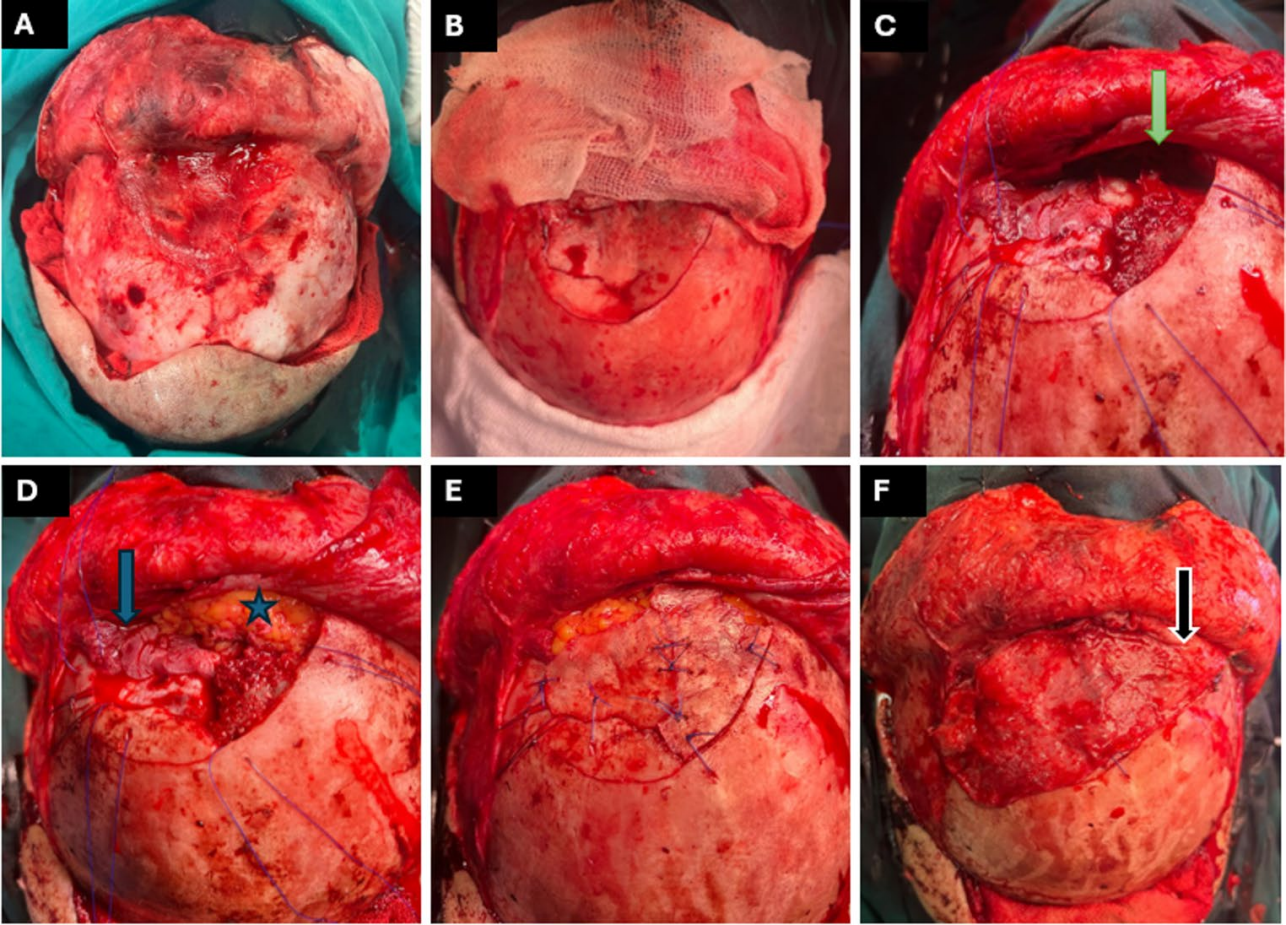
17-year-old boy after motor-vehicle collision. **A**. bicoronal incision; **B**. after harvesting a PCF split and pedicled bilaterally on superficial temporal arteries (central pedicle was torn by the fracture); **C**. posterior frontal sinus table is removed and sinus mucosa is stripped (cranialization) (green arrow points to frontal sinus ostium), **D**. the left half of the PCF (blue arrow) is used for ACF reconstruction and abdominal fat (blue star) is used for frontal sinus ostium obliteration; **E.**fractured segments stitched and craniotomy fixed in place; **F**. right half of PCF (black arrow) is used for coverage of the reduced bone

A 17-year-old male patient had a motor car accident and was presented to the ER with a depressed frontal fracture (both tables) with obliterated NFOT, as detected from his CT scans. Following stabilization of his condition, he underwent open reduction through a bicoronal incision under general anesthesia. After harvesting the PCF, the fractured anterior table was exteriorized while the comminuted/depressed posterior table segments were taken out (cranialization), and the dura was explored and stitched at the cut points. The PCF, pedicled on the supratrochlear/supraorbital bundle, was cut into two halves, where the left side was used as a Chinese carpet for reconstruction of the ACF and to support the torn dura. Since the NFOT was occluded, the anterior table sinus mucosa was debrided, the frontal ostium was obliterated with gel foam soaked in Betadine, and the frontal sinus cavity was obliterated with abdominal fat. After recontouring the comminuted anterior table into a premorbid state and fixing it with 3/0 Prolene stitches, the other half of the PCF was used to cover the fracture, aiming to give a blood supply to the comminuted bone segments to enhance healing and avoid the anxiety caused by the stitched felt under the skin. The patient was followed for 7 months, yet no complications were reported (Figs. 2b and 5).

#### Case 3

**Fig. 6:**
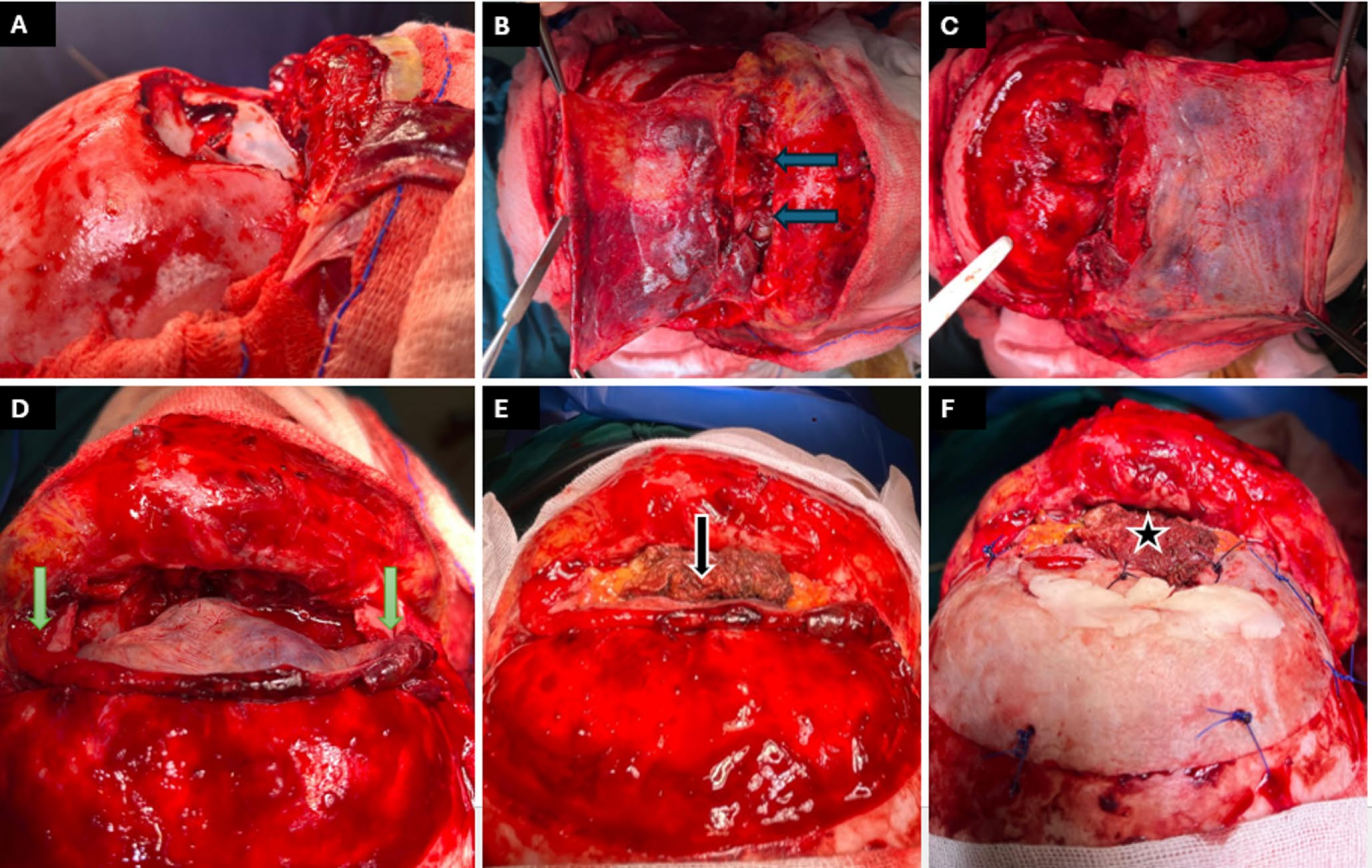
15-year-old boy, high-energy road-traffic accident. **A**.Extensively comminuted frontal sinus and superior orbital rim managed with a bicoronal approach; **B**,** C**,** D**.superficial-temporal-based (green arrows) PCF harvested as the central pedicle was torn by the fracture (blue arrows) then tucked under the frontal lobe and double folded anterior to it (Chinese Carpet in **D**) after removal of the shattered posterior table (cranialization); **E**,** F**.the sinus ostium packed with fat (black arrow) plus a temporalis-muscle flap (black star) for contouring of the forehead. Orbital reconstruction restored extra-ocular motility

A 15-year-old male had a major road traffic accident (RTA) that resulted in comminution of the frontal sinus and medial and superior orbital walls bilaterally. Ophthalmological consultation reported the limitation of upward gaze in both eyes with a visual acuity of 1/60. His general status improved after 2 days of ICU admission, and he was prepared for surgery. In a multidisciplinary collaboration, neurosurgeons started by performing a bicoronal incision and raising the PCF. A severely depressed frontal sinus caused a major tear in the overlying flap with evident trauma to the supratrochlear/supraorbital bundle, so the flap was pedicled mainly on the superficial temporal vessels. A craniotomy was raised, as the sharply angulated bony spicules were extremely difficult to manipulate and there was a high possibility of tearing the dura. Then, the PCF was gently inserted onto the ACF floor after reducing the orbital roof. The frontal sinus posterior table was totally removed (cranialized), and the otolaryngologists-maxillofacial surgeon obliterated the sinus cavity with abdominal fat and a temporalis muscle flap to replace the deficient segments of the anterior frontal table and reach a reasonable contour. Finally, the ophthalmologist operated on the superior oblique complex to avoid postoperative diplopia. No CSF leak was detected in the 4-month postoperative period (Figs. 2c and 6).

## Discussion

The frontal sinus is one of the most challenging paranasal sinuses to operate on; that is why many techniques have been described to approach it. Most frontal sinus fractures are a result of high-velocity blunt force trauma and typically include accompanying facial and neurological damage. There is still a debate regarding the best approach for managing a frontal sinus fracture. However, the majority of surgeons believe that surgical exploration is required in cases of displacement of the anterior table resulting in a deformity of the forehead, involvement of the NFOT, and displacement of the posterior table accompanied by a dural tear and CSF leakage [9].

Conventional approaches for frontal sinus fractures include transverse forehead or vertical incisions directly through a laceration wound, bilateral brow–glabella or butterfly incisions, or bicoronal incisions. The approach depends mainly on the type (closed or open) and severity of the fractures [10, 11]. The bicoronal incision, with its well-hidden nature and excellent exposure to the fracture site, has become a workhorse for frontal sinus fracture repair. But there are some complications associated with this procedure, such as an extended scalp scar, alopecia, scalp paresthesia, increased blood loss, and temporal hollowing. These drawbacks present problems for patients with uncomplicated fractures [13, 14].

Selecting appropriate reconstructive materials and techniques requires careful consideration of various factors, including defect size, location, patient age, comorbidities, and the surgeon’s technical skills and experience. Based on these factors, one can tailor the reconstruction approach for each patient [15–17]. In the current study, we describe our 10 years’ experience of managing such cases and present an illustrative algorithm of how to manage each case based on their clinical and radiologic findings.

Certain criteria describe an ideal reconstructive material. It should be chemically inert, biocompatible, nonallergenic, noncarcinogenic, cost-effective, sterilizable, easy to handle, and preferably radiopaque [18]. Lakhani et al. emphasized the use of titanium mesh as an option for the repair of severely comminuted fractures of the frontal sinus with excellent results [19].

Reconstructing ACF floor fractures presents another challenge because it requires free or vascularized tissue transfer and securing the optimal placement of these tissues. Small defects are often closed using free tissue grafts, a temporoparietal fascia flap (TPFF), or a temporalis muscle flap. The TPFF could be advanced to ACF defects through a subfrontal epidural corridor or using the endonasal transpterygoid approach. Overall, the TPFF is one of the most versatile reconstructive options, given its reliable vascularity, pliability, ability to conform to concavities, and wide arc of pedicle rotation [20–23].

Among other vascularized regional flaps is the pericranial flap (PCF), originally described by Wolfe in 1978, that continues to be widely used for reconstructing medium to large central ACF defects [13, 15]. Surgeons recommend the PCF as a safe and effective material for reconstruction. The PCF is composed of the periosteum of the scalp and the loose connective tissue that covers it. The flap has a substantial blood supply, receiving contributions from the supraorbital, supratrochlear, and superficial temporal vessels. The high blood supply of this tissue enables flexibility in design: the flap can be unilateral or bilateral, and it can be based either anteriorly or laterally. The PCF can be easily and quickly obtained, and its application in frontal sinus surgery eliminates the need for an additional donor site due to its presence inside the surgical field. In cases where a thicker flap is desired, the galea can also be incorporated into the flap [10, 11, 16].

Most reports describe flap elevation after a traditional bicoronal incision, although other routes of access may include pretrichial, trichophytic, through preexisting lacerations, and even the midforehead approach for shorter flaps [24, 25]. Zanation et al. introduced an innovative technique for harvesting and placing the PCF through a bony defect in the nasion, which is used to reconstruct defects following endonasal anterior skull base (ASB) resection [26].

Over the past couple of decades, the preference for approaches has drastically shifted toward minimally invasive ones. Consequently, the corridors that transpose a vascularized flap have also evolved. The endoscopically assisted approach achieves nearly the same benefits as a bicoronal incision with only two small incisions. This technique offers more direct observation, prevention of damage to neurovascular pedicles, minimum manipulation of tissues, and a superior aesthetic result with a shorter hospitalization period due to the smaller surgical incision and improved recovery [9].

Nevertheless, not all frontal sinus fractures are appropriate for this technique. Endoscopically assisted surgery is not suitable for a displaced posterior table fracture with evidence of a dural tear, as well as extensive bone fractures. In such circumstances, the dural repair and reduction of a compressed cranial fracture through craniotomy or cranialization need more complicated procedures, which are more effectively performed with a conventional bicoronal incision [9].

Conclusively, the keys to surgical success include adequate preoperative evaluation, intraoperative multidisciplinary collaboration, exposure and visualization of the entire frontal bone fracture, meticulous flap elevation and harvesting, and close postoperative follow up.

To the best of our knowledge, the present report is one of the largest studies that focuses on frontal sinus fractures and illustrates an approach on how to deal with different case scenarios. Moreover, the study shows various techniques of using the PCF as reconstruction materials for ACF floor coverage within a reasonable follow up period. Yet, Larger-scale series with longer follow-ups are needed to further clarify the advantages of the PCF, especially in the setting of extensive frontal trauma, to gain a conclusive idea of its benefits in reducing the risk of CSF leak and perioperative infections.

One limitation of this study is the lack of a standardized method for evaluating forehead projection, as management was individualized according to fracture characteristics (simple versus comminuted), the viability of the flap pedicle (supratrochlear/supraorbital or superficial temporal), and the extent of residual bony depression following reduction. Although a mean change in projection of 3.4 ± 1.7 mm might not be aesthetically perceptible in some cases, the authors consider radiological perfection to be an unrealistic objective. Surgical intervention in this critical anatomical region should be confined to restoring function and correcting deformity while minimizing the risk of potential complications. Another limitation is the low incidence of complications, which precluded meaningful subgroup analysis to determine whether complication rates differed between patients with isolated anterior table fractures and those with combined anterior and posterior table fractures.

## Conclusion

Selecting the appropriate surgical approach and the reconstructive material should be tailored to each case of depressed frontal fracture. The PCF stands as a favorable option with reliable and diverse vascularity, pliability, versatility, and satisfactory postoperative outcomes with no need for a second donor site morbidity. However, it is necessary to conduct studies with larger sample sizes and longer follow-up periods to reach a more solid and generalizable conclusion.

## Data Availability

No datasets were generated or analysed during the current study.
